# Genome-wide identification of cyclic nucleotide-gated channel gene family in *Solanum tuberosum* and silencing of *StCNGC2* provides resistance to *Pectobacterium carotovorum*

**DOI:** 10.3389/fpls.2025.1614191

**Published:** 2025-07-15

**Authors:** Kaile Sun, Shuai Liu, Huipo Mao, Qianqian Zha, Han Liu, Shunshan Shen, Evert Jacobsen, Richard G. F. Visser, Yuling Bai, Chengwei Li, Zhiqi Jia, Geng Meng, Yawen Shen

**Affiliations:** ^1^ College of Horticulture, Henan Agricultural University, Zhengzhou, Henan, China; ^2^ Henan International Joint Laboratory of Horticultural Plant Biology, Henan Agricultural University, Zhengzhou, Henan, China; ^3^ College of Plant Protection, Henan Agricultural University, Zhengzhou, Henan, China; ^4^ Plant Breeding, Wageningen University and Research, Wageningen, Netherlands

**Keywords:** calcium signaling, plant immunity, pathogenesis-related proteins, blackleg resistance, potato

## Abstract

Cyclic nucleotide-gated ion channel (*CNGC*) genes play vital roles in plant growth, development, and responses to both biotic and abiotic stresses. However, the current research on CNGCs in potato (Solanum tuberosum) remain largely uncharacterized. Blackleg disease is one of the most devastating diseases worldwide, causing severe yield losses. Understanding the role of the *StCNGC* gene family in blackleg resistance is therefore of significant importance. In this study, we identified 11 *StCNGC* genes in the potato genome and conducted phylogenetic analysis, gene structure characterization, and conserved motif prediction. Expression patterns were examined in different tissues and under stress conditions. The identified StCNGCs were classified into five groups, and showed conserved gene structures and motifs within groups. Most StCNGCs were induced under biotic stress conditions. Notably, silencing *StCNGC2* conferred resistance to blackleg disease and resulted in the upregulation the pathogenesis-related marker gene StPR1. Together, these findings suggest that *StCNGC2* plays a crucial role in potato defense against blackleg disease and provide a foundation for further functional studies of the *StCNGC* gene family.

## Introduction

Calcium ions (Ca^2+^) serve as critical secondary messengers in plant signaling pathways, mediating responses to developmental cues and environmental stimuli (He et al., 2018; Yang et al., 2021). Ca^2+^ transport across membranes is facilitated by channels and carriers, with channel proteins as the main path for rapid Ca^2+^ transport crossing the membrane. As such, calcium channels are essential for generating calcium signals (Wang et al., 2021a).

Cyclic nucleotide-gated channels (CNGCs) are ligand-gated Ca^2+^ channels that are essential for signal transduction and cellular homeostasis (Jarratt-Barnham et al., 2021; Wang et al., 2024). They possess distinctive structural features, comprising six transmembrane helices (S1–S6) with a pore-forming segment located between S5 and S6, and a cyclic nucleotide (cNMP)-binding domain (CNBD) and a calmodulin (CaM) binding domain (CaMBD) at the C-terminus (Kaplan et al., 2007; Jarratt-Barnham et al., 2021). CNGCs can be activated by cNMP binding and inhibited by Ca^2+^/CaM binding (Trudeau and Zagotta, 2002). CaM binds to isoleucine glutamine (IQ) motifs in a Ca^2+^-dependent manner (Fischer et al., 2013). The CNBD contains a hinge region and a phosphate-binding cassette (PBC) (Jarratt-Barnham et al., 2021). PBC interacts with cAMP ribose-phosphate, while the flexible helix, referred to as the “hinge”, interacts with the PBC (Young and Krougliak, 2004; James and Zagotta, 2018). Owing to their structural uniqueness, CNGCs are candidates for ligand-gated/voltage-independent/cation transporters, which are significant in both plant development/growth regulation and biotic/abiotic stress responses (Kaplan et al., 2007; Moeder et al., 2011).

Multiple *CNGC* genes have been identified across different plant species, for instance, 20 members in *Arabidopsis thaliana* (Mäser et al., 2001), 47 in *Triticum aestivum* (Guo et al., 2018), 18 in *Solanum lycopersicum* (Saand et al., 2015), 16 in *Oryza sativa* (Wang et al., 2023), 26 in *Brassica oleracea* (Kakar et al., 2017), 29 in *Solanum melongena* (Jiang et al., 2023), 29 in *Brassica rapa* (Baloch et al., 2021), 20 in *Malus domestica* (Qiu et al., 2024), 35 in *Nicotiana tabacum* (Nawaz et al., 2019), 15 in *Ziziphus jujuba* (Wang et al., 2020), 16 in *Saccharum* sp*ontaneum* (Zhang et al., 2023a), 43 in *Mangifera indica* (Zhang et al., 2023b), 12 in *Zea mays* (Hao and Qiao, 2018), and 21 in *Pyrus* spp (Chen et al., 2015).

In *Arabidopsis*, *CNGC* genes were divided into 5 groups. Members from each of the groups have been reported to be involved in developmental regulation and stress resistance (Mäser et al., 2001; Dietrich et al., 2020; Tipper et al., 2023). For instance, the AtCNGC2–AtCNGC4 channel complex is known to be negatively regulated by calmodulins (CaMs) and positively regulated by the immune kinase BOTRYTIS-INDUCED KINASE 1 (BIK1). In the resting state, CaMs suppress channel activity, while upon pathogen detection, BIK1 phosphorylates and activates the channel, promoting Ca^2+^ influx for pattern-triggered immunity activation (Zhang et al., 2024b). The *AtCNGC2* (*DND1*) mutant is resistant to a variety of pathogens, including the bacterium *P. syringae*, the fungal pathogens *B. cinerea* and *A. brassicicola*, and the oomycete *H. arabidopsidis* (Jurkowski et al., 2004; Young and Krougliak, 2004; Su’udi et al., 2011). The chimeric gene *Arabidopsis CNGC11/12* activates resistance responses toward multiple pathogens (such as *Hyaloperonospora parasitica* and *Phytophthora parasitica*) (Yoshioka et al., 2001, 2006). Similarly, *AtCNGC19* is induced by *P. indica* infection, and it regulates the synthesis and accumulation of indole glucosinolates, and further increases plant pathogen resistance (Jogawat et al., 2020).

Recent studies in other plant species have further highlighted the role of CNGCs in stress resistance. For instance, knocking down *CNGC2* orthologs in tomato and potato markedly reduced their vulnerability to fungal infections responsible for powdery mildew, late blight, and gray mold (Sun et al., 2016a, 2016b, 2017). Similarly, mutations in the apple *MdCNGC2* gene resulted in constitutive accumulation of salicylic acid (SA) and *pathogenesis-related protein 1* (*MdPR1*) in apple callus, thereby restricting the spread of *B. dothidea* (Zhou et al., 2020). In *Z. jujuba*, ZjCNGC2 was shown to interact with mitogen-activated protein kinase 4 (MAPK4), suggesting its involvement in cold stress responses via the MAPK signaling cascade (Wang et al., 2020).

Potato (*Solanum tuberosum*, auto-tetraploid, 2n=4x=48) is the fourth most important food crop worldwide (Hou et al., 2019; Fan et al., 2022), following maize (*Zea mays*), rice (*Oryza sativa*), and wheat (*Triticum aestivum*) in 2019 (https://www.fao.org/home/en/). Despite its significance, a substantial amount of potato yield is lost per year due to viral, bacterial, or fungal diseases and pest attacks (Czajkowski et al., 2011; Zhang et al., 2019; Wu et al., 2020). Among these, soft rot and blackleg, caused by the bacterial pathogen *Pectobacterium carotovorum*, are particularly devastating. These diseases present in distinct forms, with stem infections referred to as blackleg and tuber infections as soft rot (Pérombelon, 2002). Therefore, there is an urgent need for sustainable and environmentally friendly strategies to control crop diseases (Zhu et al., 2024). Susceptibility (S) genes are plant genes exploited by pathogens to promote disease development. Mutations in S genes are typically recessively inherited and are considered a durable strategy to confer broad-spectrum resistance in plants (Garcia-Ruiz et al., 2021; Koseoglou et al., 2022; Gao et al., 2024). As previously noted, CNGCs have been identified as potential S genes in several plant species. Despite their importance, studies on the *CNGC* gene family in potato remain limited, particularly regarding their role in biotic stress resistance.

In this study, we identified 11 *StCNGC* genes and analyzed their structural features, phylogenetic relationships, and expression patterns under stress conditions. Further functional validation through silencing of *StCNGC2* revealed its involvement in enhancing resistance to *P. carotovorum*. These findings contribute to our understanding of *StCNGCs* in improving disease resistance in this critical crop.

## Methods

### Identification of *StCNGC* family *genes*

To identify *CNGCs* in potato, 20 *Arabidopsis* CNGC protein sequences were retrieved from TAIR (Mäser et al., 2001). We employed these sequences as queries to search against the potato genome sequences downloaded from the Potato Genome Sequencing Consortium database (PGSC, version 6.1). A cut-off of E value < 10^–5^ was applied to ensure the reliability of the sequences. Meanwhile, the HMM (Hidden Markov Model) files cNMP_binding.hmm (Cyclic nucleotide-binding domain, PF00027) and Ion_trans.hmm (Ion transport protein, PF00520) were downloaded from the Pfam database (Paysan-Lafosse et al., 2025). Simple hmmsearch (Potter et al., 2018) from TBtools (Chen et al., 2023) was carried out to search for CNGC proteins in potato. The protein sequences obtained from BLASTp and hmmsearch methods were then combined, with redundant sequences removed. To check the presence of CNBD and ion transport domains, we submitted these protein sequences to the SMART program (Schultz, 2000), InterPro (Blum et al., 2024), and NCBI-CDD database (Marchler-Bauer et al., 2017). Proteins without CNBD and ion transport domains were excluded from further analysis. The same approach was employed to identify CNGCs in four Gramineae crops (*Z. mays*, *T. aestivum*, *O. sativa*, and *S. bicolor*) and one nightshade member (*S. lycopersicum*).

### Analysis of physicochemical properties and chromosomal location

ExPaSy (Duvaud et al., 2021) was used to calculate the molecular weight (MW) and isoelectric point (pI) of the proteins. Chromosomal distribution of *StCNGCs* and genetic sequence data were obtained from a BLASTn search of the EnsemblPlant database (Yates et al., 2022). The genomic locations of *StCNGC* genes were visualized on chromosomes using MapChart software based on their genome coordinates.

### Gene structure, conserved motifs and promoter *cis*-acting elements analysis

The exon-intron structures were analyzed using the online program GSDS (Hu et al., 2015), utilizing the coding sequences (CDS) and whole gene sequences from the *StCNGC* genes. The conserved motifs were identified using the online program MEME (Bailey et al., 2009), with parameters set for motif widths ranging from 6 to 200 bp and a maximum of 12 motifs. The 1500 bp sequence upstream of the promoter region of each *StCNGC* was identified by searching EnsemblPlants (SolTub_3.0), and was then analyzed using PlantCARE (Lescot, 2002) for the *cis*-acting elements analysis. The obtained data were then visualized by using TBtools (v2.146) software (Chen et al., 2023).

### Analysis of phylogenetic relationships, gene duplication events

A multiple sequence alignment of the amino acid sequences was performed using DNAMAN software (version 6.0). The whole amino acid sequences were aligned using the ClustalW algorithm, and a phylogenetic tree was generated with MEGA software (version 7) (Kumar et al., 2016), employing the neighbour-joining (NJ) approach with 1000 bootstrap iterations (Bi et al., 2024). Gene duplication analysis of the *CNGC* genes was explored by plotting intraspecies covariance maps using the TBtools (v2.146) software built-in Dual Synteny Plot program function (Chen et al., 2023).

### Plant abiotic stress treatments

Three-week-old *in vitro-*propagated potato plantlets were transplanted into soil and grown in a greenhouse under controlled conditions (25 ± 2°C, 75% relative humidity, and a 16:8 h light/dark photoperiod). Potato derivatives cv. Désirée was used as a non-transgenic control. *RNAi::StCNGC2* transgenic lines *#5* and *#17*, corresponding to *DND1A-5*(*+*) and *DND1A-17*(*+*), were obtained from a previous study. They exhibit significantly reduced *StCNGC2* expression (Sun et al., 2016a).

For abiotic stress treatments, potato plants grown in the greenhouse for 4–6 weeks were exposed to the following conditions: drought stress by withholding water for 8 days, heat stress by maintaining greenhouse temperature at 42°C for 2 d, and cold stress by incubating plants at 4°C for 5 d. Potato leaves were collected at the end of each treatment. All materials were snap-frozen in liquid nitrogen and stored at − 80°C for subsequent expression analyses. Three biological replicates (four plants per replicate) were examined for each treatment.

### Pathogen infection assays

Four–six-week-old potato plants with fully developed composite leaves were infected with *Phytophthora infestans, Botrytis cinerea*, and *Pectobacterium carotovorum* to assess pathogen response. Detached leaves were infected with *P. infestans* strain 88069, following the method described by Vleeshouwers et al. (1999). Leaf samples were collected at 48 hours post-inoculation (hpi). For the *B. cinerea* infection, leaflets were inoculated with strain B05.10 at a density of 3×10^5^ spores/ml, and the inoculation method was referred to Sun et al. (2017). Leaf samples were collected at 48 hpi. For the bacterial assay, *P. carotovora subsp. brasiliense* strain 212 was prepared at a concentration of 1×10^3^ cfu/ml, and the inoculations were performed as described by Rietman et al. (2014). Stem samples were collected at 46 hpi. An equal volume of autoclave ddH_2_O was used as mock treatment following the same procedure as for pathogen inoculation. All plant samples were immediately frozen in liquid nitrogen and stored for the following gene expression analyses. For each treatment, three replicates (four plants per replicate) were examined.

### Analysis of tissue-specific gene expression

Transcriptome data were acquired from the Spud DB website (http://solanaceae.plantbiology.msu.edu/index.shtml) to examine the expression patterns of *StCNGC* genes in different tissues. Five different organs were included in the analysis, namely, leaves, flowers, roots, stems, and tubers. Gene expression levels were quantified as fragments per kilobase per million mapped reads (FPKM) (**Supplementary Table S6**). Raw FPKM values were normalized by the logarithmic method, and a heat map was generated with TBtools software (Chen et al., 2023) to visualize the expression levels.

Additionally, leaves, flowers, roots, stems and tubers were also collected from potato cv. Désirée plants for total RNA extraction and validation of expression patterns via RT-qPCR analysis.

### Anatomical analysis of stem structures

To examine the infection of *P. carotovora*, stem cross-sections were collected 1 cm above the inoculation site at 0, 12, 24, 36, and 46 hpi using sharp scalpels. Samples for each genotype were observed using a bright-field binocular microscope (Olympus SZX16, Japan). Images were captured using a microscope-mounted digital camera (TOUPCAM™, Germany) with consistent settings across all observations. For each time point, at least twelve plants were analyzed.

### RNA isolation and gene expression analysis

Total RNA was extracted using an RNA isolation kit (Huayueyang, China), following the manufacturer’s protocol. The concentration and quality of RNA were assessed using a Nanodrop 2000 spectrophotometer (Thermo Scientific, China). First-strand cDNA was synthesized using a cDNA synthesis kit (Monad, MonScript™ RTIII AII-in-One Mix with dsDNase, China).

Quantitative real-time PCR (RT-qPCR) was conducted on a C1000™ Thermal Cycler PCR system (Bio-Rad) by using MonAmp™ SYBR^®^ Green qPCR Mix (Monad, None ROX). The potato *StEF1a* (Sotub06g010680) served as an internal control. The comparative gene expression level was determined utilizing the 2 ^-ΔΔCT^ method (Livak and Schmittgen, 2001). Three technical replicates were included for each reaction, and three biological replicates were included for each sample. Primer sequences are listed in **Supplementary Table S7**.

## Results

### Identification of *StCNGC* gene family in potato

To identify the *CNGC* gene family of potato, BLASTP was performed based on the sequence of the 20 CNGC members of *Arabidopsis thaliana*. Meanwhile, Hidden Markov models (HMMs) for the cNMP_binding domain (PF00027) and the Ion_trans domain (PF00520) were applied using hmmsearch against the potato genome. Afterwards, SMART, InterPro, PROSITE conserved domain search tools, and NCBI Conserved Domain Data (CDD) were employed to confirm the presence of CNBD and ion transport domains in the putative proteins. Domain composition analyses revealed that some candidate genes carried potassium channel AKT/KAT domains (Shaker type) (Su et al., 2001). Therefore, the remaining 11 genes, including CNBD and ion transport domains, and no additional potassium channel domains, were designated as *StCNGC* genes.

**Figure 1:**
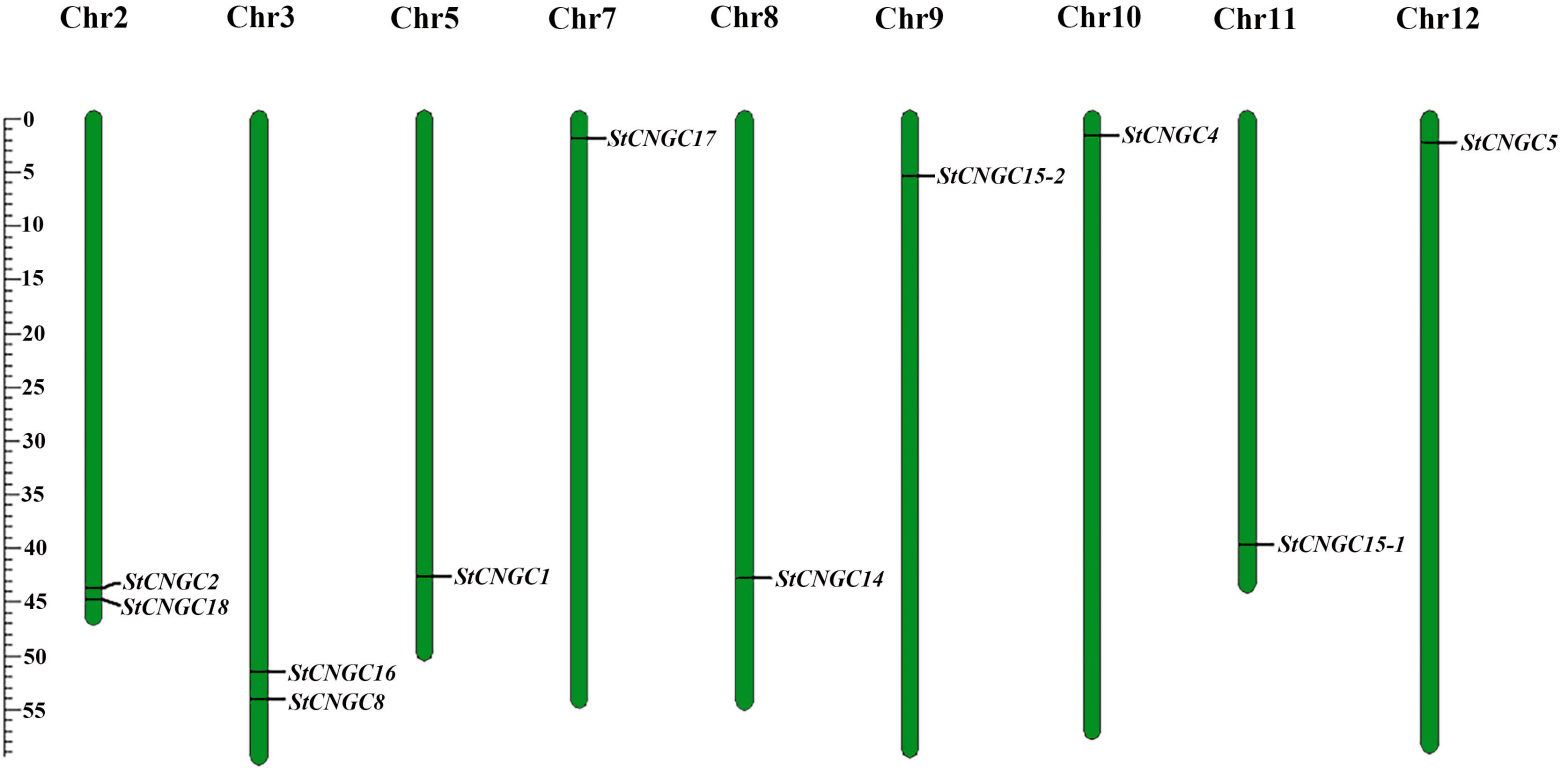
Chromosomal distribution of potato *CNGCs.* Each *CNGC* member was mapped on its physical chromosome location. The chromosome number (Chr02-Chr12) is indicated at the top. Only chromosomes containing *StCNGC* genes are represented in this figure.

Chromosomal mapping revealed their distribution across nine of the 12 potato chromosomes (**Figure 1**). There were two *StCNGC* genes on Chr 2 and Chr 3 and one *StCNGC* gene on each of the other chromosomes. No genes were found on Chr 1, Chr 4 and Chr 6. The identified StCNGC protein length ranges from 659 (StCNGC15-2) to 727 (StCNGC14) amino acids (aa) in length, with molecular weights (MWs) of 76036.04 to 83853.48 Da. Most StCNGC proteins (except for StCNGC8 and StCNGC18) had Isoelectric point (pI) values above 8.0.

### Sequence alignment and phylogenetic analysis

**Figure 2:**
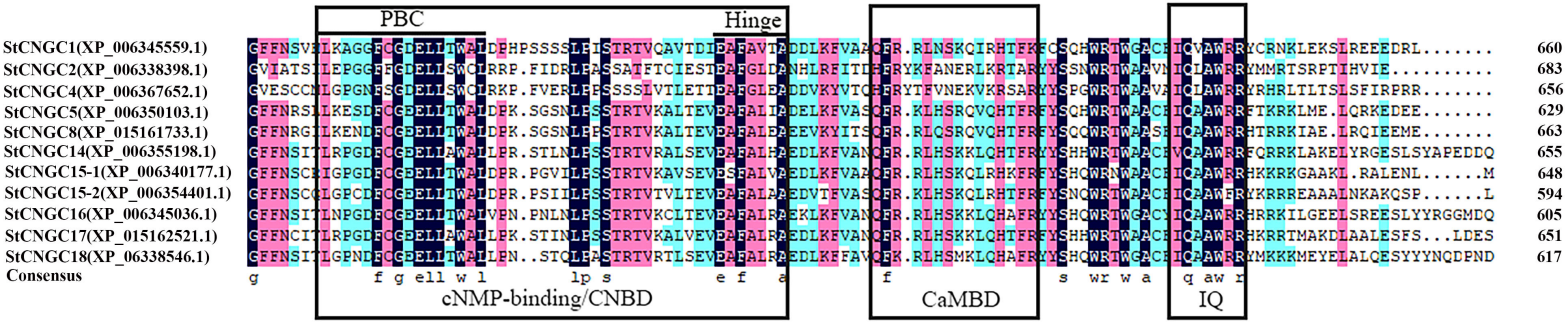
Conserved amino acids and multiple sequence alignment of the StCNGC proteins. Sequences were displayed by the DNAMAN software. The StCNGC conserved sequences were marked with a purple background for an amino acid identity greater than 75% and a light blue background for an amino acid identity greater than 50%. The family-specific conserved motifs and active sites are indicated.

Sequence alignment of StCNGCs has revealed a variable number of transmembrane helices, ranging from 3 to 7 (**Supplementary Table S1**), as well as highly conserved CNBD and CaMBD, and isoleucine–glutamine (IQ) motifs (**Figure 2**). Within the CNBD domain, the two most conserved regions are the PBC and the adjacent hinge region (**Figure 2**). The consensus PBC motif was identified to be: [L]-X(4)-[F]-X-[G]-[DE]-[E]-[L]-[L]-X-[W]-[AC]-[L]-X(6,7,8)-[L]-[P]-X-[S]-[TS]-X-[TS]-X(7)-[E]-[AS]-[F]-[AG]-[VL]-X-[A] (X denotes any residue). The CaMBD motif, [H]-[F]-[R]-[Y]-X-[F]-X-[N]-[E]-X(2)-[K]-[R]-X-[A]-[R], was not well conserved, while the IQ motif [I]-[Q]-X-[A]-[W]-[RF]-[R] was relatively conserved among the StCNGC proteins.

To assess the evolutionary relationships of the StCNGC proteins, complete protein sequences of 91 CNGCs from seven angiosperm species were analyzed phylogenetically using MEGA software. These CNGC proteins included 20 sequences from *Arabidopsis*, 11 from potato, 13 from tomato, 13 from corn, 14 from rice, 8 from wheat, and 12 from sorghum (**Supplementary Table S2**). The resulting phylogenetic tree clustered the CNGC proteins into five distinct groups (Groups I, II, III, IVa, and IVb), following the established *Arabidopsis* CNGC classification. Group III emerged as the largest with 34 genes, including six potato *CNGC* genes (*StCNGC14*, *StCNGC15-1*, *StCNGC16*, *StCNGC17*, and *StCNGC18*). Group II and Group IVb contained 20 and 13 genes, respectively, each with two potato CNGC members. Group I contained only one potato *CNGC* gene (*StCNGC1*) and no *StCNGC* was assigned to Group IVa.

### Gene structure analysis and motif identification

**Figure 3:**
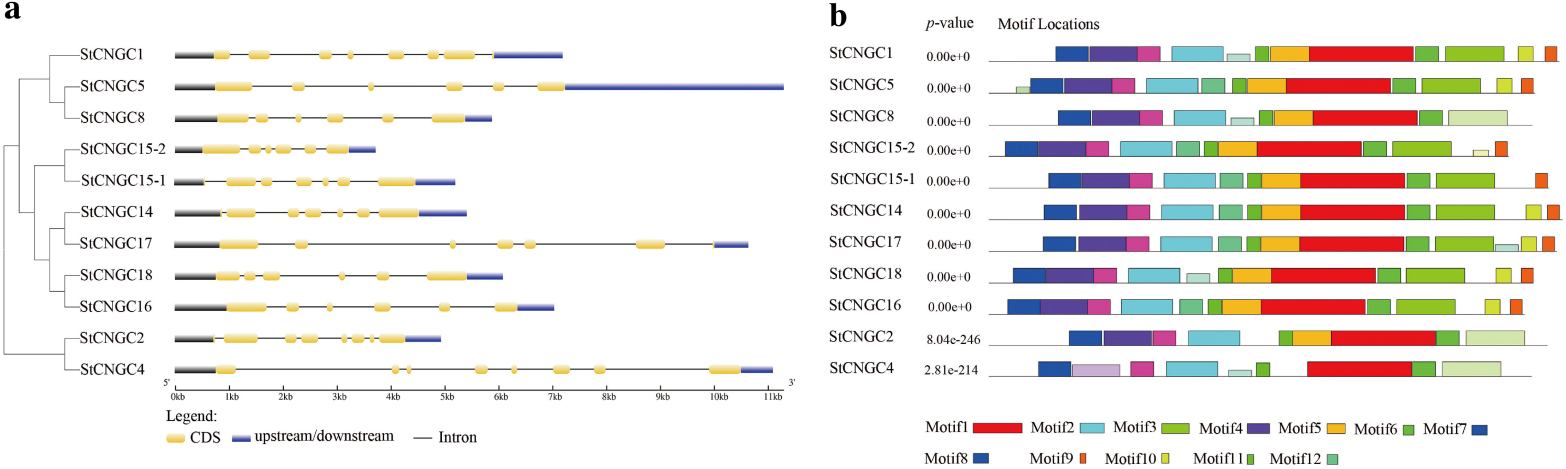
Schematic structure of *CNGC* genes in potato. **(a)** Gene structural analysis of potato *CNGCs*. Introns and exons are represented by black lines and yellow boxes, respectively. The untranslated regions (UTRs) are indicated by blue boxes. The sizes of the exons and introns can be estimated using the scale at the bottom. **(b)** Putative protein motifs of each potato StCNGCs by MEME. Conserved motifs (1–12) are represented by different colored boxes while non-conserved sequences are shown by gray lines.

We also conducted exon-intron structure analyses based on the corresponding genome and coding sequences. The number of exons in the potato CNGC family varied from 6 to 8 (**Figure 3**). To further elucidate the structural and functional properties of the StCNGC proteins, conserved motifs were identified using MEME (Bailey et al., 2009). Twelve putative motifs, designated as motifs 1 to 12, were identified in the StCNGC family (**Figure 3**, **Supplementary Table S3**). The relative positions and arrangements of these motifs varied across the five phylogenetic groups, forming distinct motif patterns (**Figure 3**). All the StCNGCs contained motifs 1–8 and motif 11, representing the core conserved motifs of the StCNGC family. Motif 1 is the CNBD located at the C-terminal region, while motif 12 corresponds to the CaMBD and the IQ domain. Furthermore, motifs 2, and 4–8 correspond to the transmembrane domain in the N-terminal region. Interestingly, some motifs exhibited gene-specific losses. For instance, motif 9 was not found in *StCNGC2*, *StCNGC4*, and *StCNGC8*, while motif 10 was not found in *StCNGC2*, *StCNGC4*, *StCNGC8*, and *StCNGC15-1*. Motifs 3, 9, 10, and 11 are responsible for unknown functions.

### Covariance analysis of the *StCNGCs*

**Figure 4:**
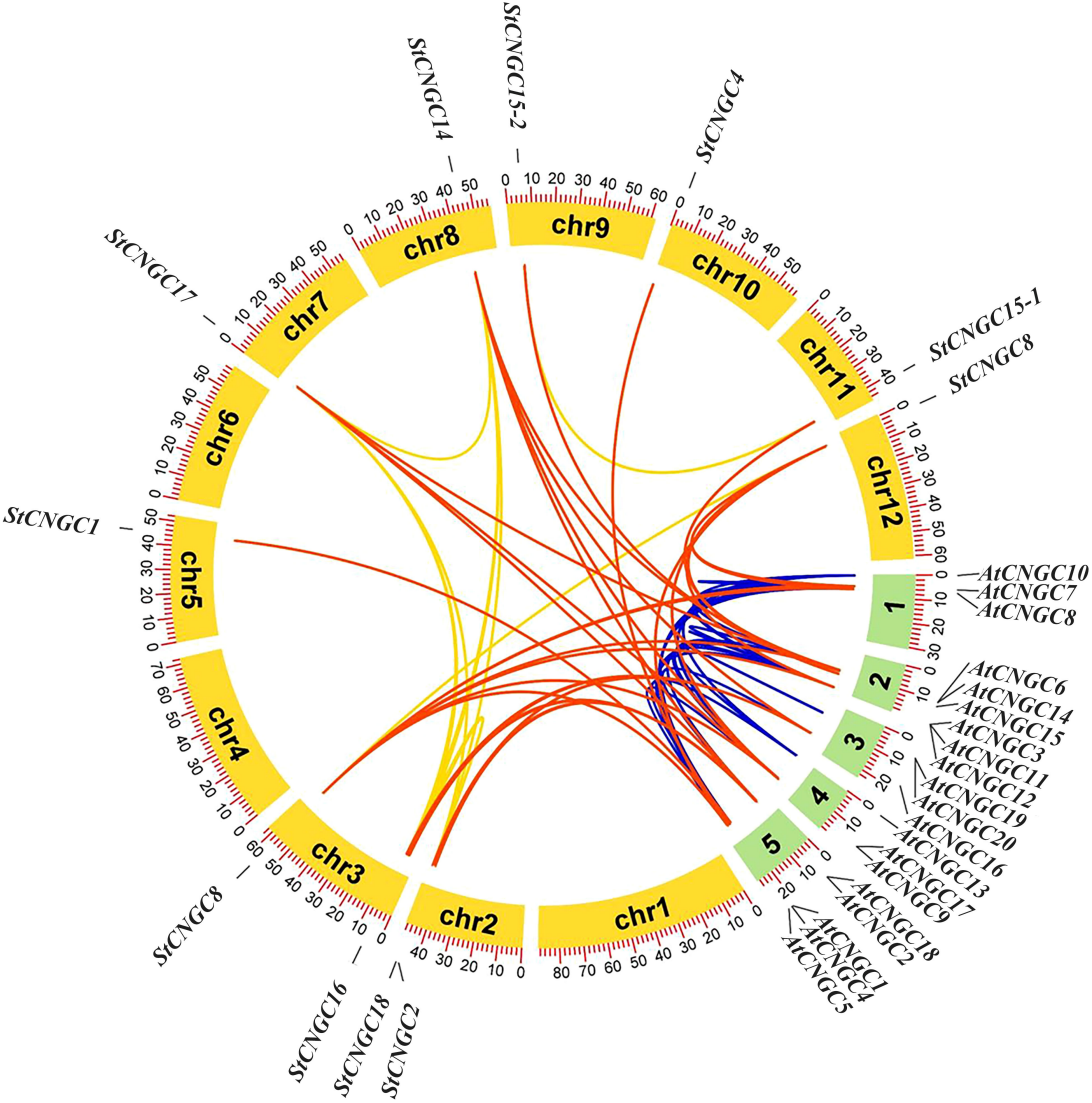
Homology analysis of *CNGCs* between potato and *Arabidopsis* genomes. Yellow arcs represent potato chromosomes and green arcs represent *Arabidopsis* chromosomes. Red, blue, and golden lines indicate homologous gene pairs between potato and *Arabidopsis*.

Collinear analysis of potato and *Arabidopsis CNGCs* revealed that 28 segmental duplication gene pairs were detected between *Arabidopsis* and potato, with amino acid sequence similarity greater than 60%. The potato genome contained 8 segmental duplication gene pairs (**Figure 4**). The allocation of segmental duplication genes across chromosomes was unequal, with two genes located on chromosome 2, two on chromosome 3, and one gene present on chromosomes 5, 7, 8, 9, 10, 11, and 12. Overall, the *CNGC* gene family in potato exhibited notable collinearity with *Arabidopsis*.

### *Cis*-acting elements analysis of the *StCNGCs*

To examine the potential *cis*-elements participating in the transcriptional regulation of *StCNGCs*, a 1.5 kb promoter region upstream of each *StCNGC* coding sequence was investigated in PlantCARE database. As summarized in **Table 1**, the *cis*-acting elements in the promoter regions of plant *StCNGCs* were highly diverse. A total of 78 types of *cis*-acting elements were detected, including 33 with unknown functions. The predominant elements in the *StCNGC* promoter regions were light-responsive elements, comprising 16 distinct types (**Supplementary Table S4**). The number of *cis*-elements per promoter ranged from a minimum of 15 in *StCNGC5* to a maximum of 68 in *StCNGC18* (**Table 1**). Eight types of hormone response elements were detected across the promoter regions, corresponding to auxin, abscisic acid, SA, gibberellin and jasmonic acid. Notably, 10 corresponding hormone response elements were identified in *StCNGC2* and *StCNGC8*, while *StCNGC5* and *StCNGC14* lacked hormone-responsive elements. Particularly, auxin *cis*-regulatory elements, including one auxin-responsive region (AuxRR) and a TGA box, were found in the promoter regions of *StCNGC8*, *StCNGC15–2* and *StCNGC17*, which indicated potential auxin regulation (**Supplementary Table S4**).

**Table 1:** Number of different *cis*-acting elements in the promoter region of each potato *StCNGC* gene.

Gene name	Development related elements	Environmental stress related elements	Hormone-responsive elements	Light-responsive elements	Promoter related elements	Site-binding related elements	Others*
*StCNGC1*	2	4	2	5	43	3	46
*StCNGC2*	0	3	10	7	60	0	45
*StCNGC4*	0	3	4	8	46	1	35
*StCNGC5*	0	4	0	6	15	2	61
*StCNGC8*	0	1	10	7	37	1	57
*StCNGC14*	0	2	0	17	48	3	52
*StCNGC15-1*	1	2	1	6	69	3	54
*StCNGC15-2*	1	1	2	1	51	0	52
*StCNGC16*	0	1	3	3	57	0	42
*StCNGC17*	0	0	2	7	49	0	52
*StCNGC18*	3	1	2	9	68	3	53

*Others means uncategorized.

In addition to hormonal regulation, environmental stress-related elements were identified in the promoter regions. Four types of stress-responsive elements were observed, with ARE elements (*cis*-acting regulatory element essential for the anaerobic induction) presented in 8 *StCNGCs* and TC-rich repeats (TC-rich repeats) detected in 5 *StCNGCs* (**Supplementary Table S4**).

Additionally, the GCN4 motif, associated with endosperm expression, was the predominant development-related element found in the promoters of three *StCNGC* genes. *StCNGC1* was the only gene containing one circadian control element, while *StCNGC18* promoter contained elements associated with palisade mesophyll cell differentiation (**Supplementary Table S4**).

### Tissue-specific expression analysis of the *StCNGC* gene family

A hierarchical clustering heatmap was constructed to examine the expression patterns of *StCNGC* genes in different plant organs (root, stem, leaf, flower, and tuber) utilizing transcriptome data from the PGSC database. Results showed that *StCNGC8*, *StCNGC16*, and *StCNGC18* had similar expression patterns, being highly expressed in flowers but nearly undetectable in other tissues. Although *StCNGC14* was expressed in all checked tissues, its expression levels remained low. While *StCNGC2*, *StCNGC4*, *StCNGC5*, and *StCNGC17* exhibited high expression across all analyzed organs, and *StCNGC2* and *StCNGC5* showed the highest expression in flowers and roots, respectively (**Supplementary Figure S1**).

**Figure 5:**
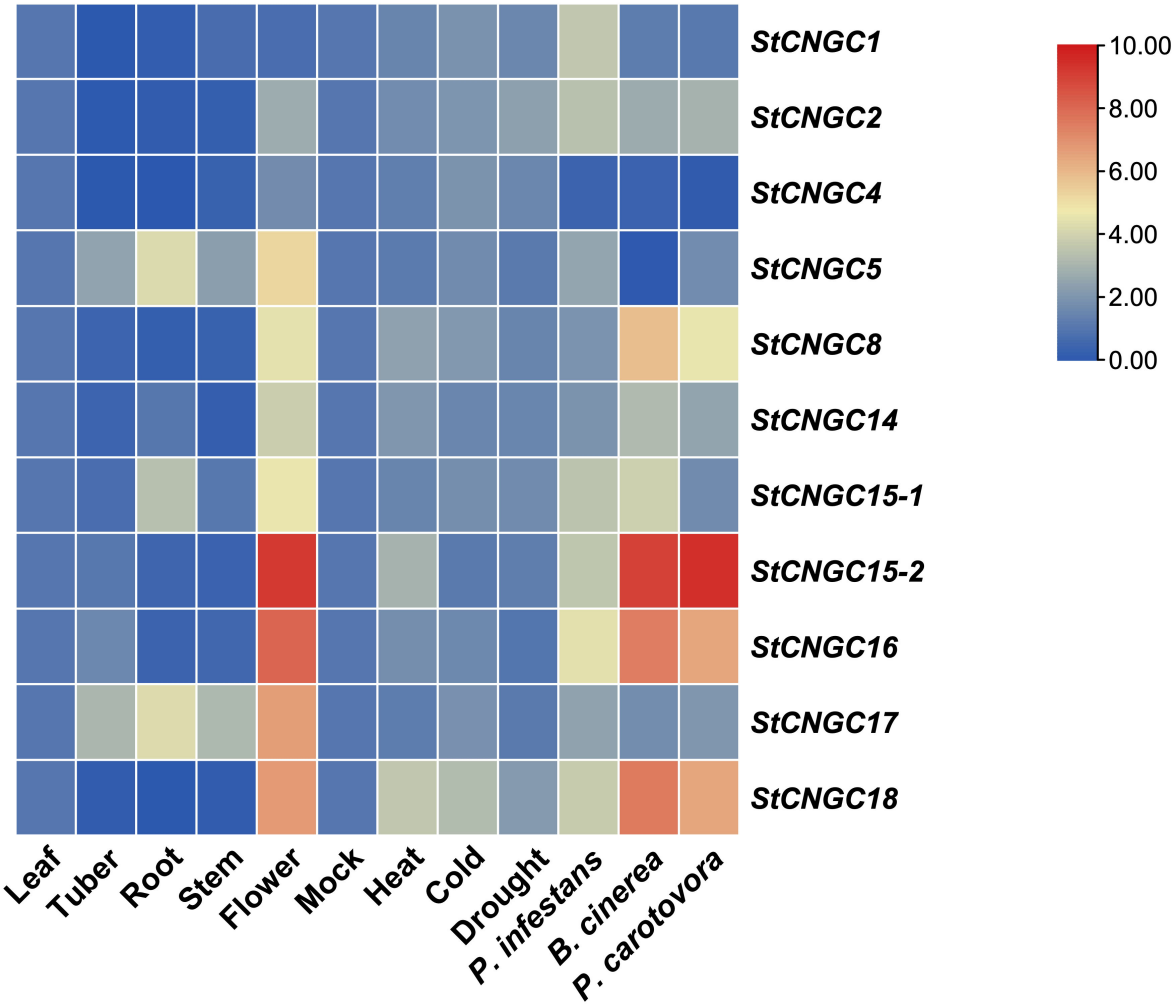
Relative expression analysis of *StCNGCs* in different tissues and under different stress conditions. For the tissue-specific analysis, leaf, tuber, root, stem, and flower samples were collected from mature cv. Désirée plants for RNA extraction and RT-qPCR analysis. For stress-response analysis, leaves (abiotic and biotic treatments) and stems (for blackleg infection only) were collected for RNA extraction and RT-qPCR analysis. The stress conditions are as follows: Heat (42°C for 2 days), Cold (4°C for 5 days), Drought (water withheld for 8 days), *Phytophthora infestans* infection for 48 h, *Botrytis cinerea* infection for 48 h (Bc/48), and *Pectobacterium carotovorum* infection for 46 h. The corresponding mock treatments included room temperature, normal watering, or ddH_2_O inoculation for the same duration as each stress condition. All stress-treated samples were compared to their respective mock controls. The mock shown in the heatmap is a collection of condition-specific controls, not a universal mock used across all experiments. The potato *StEF1a* gene was used as an internal control, and three independent samples were included in these experiments. Gene expression levels are represented as log2(Fold change), where red indicates high expression levels and blue indicates low expression levels.

To validate the transcriptome data, we further analyzed the expression of 11 *StCNGCs* in different organs of potato cv. Désirée by RT-qPCR. The results were largely consistent with the published transcriptome data (**Figure 5**, **Supplementary Table S5**): *StCNGC5* and *StCNGC17* were highly expressed in roots, with low expression in tubers and stems. Most *StCNGC*s were significantly expressed in flowers, except for *StCNGC1*, *StCNGC2*, and *StCNGC4*, which showed low expression levels, contrary to the transcriptome data findings. This discrepancy may result from the differences in sampling methods or developmental stages between this study and the previous studies.

To investigate the potential function of *StCNGCs* in response to stresses, we further analyzed their expression in potato leaf under control conditions and different stress treatments using RT-qPCR (**Figure 5**, **Supplementary Table S5**). None of the *StCNGCs* were induced under drought stress. For heat and cold stress, only *StCNGC18* showed increased expression compared to control. Notably, some *StCNGCs* were significantly induced under biotic stress conditions. For instance, the expression levels of *StCNGC1*, *StCNGC2*, *StCNGC15-1*, *StCNGC15-2*, *StCNGC16*, and *StCNGC18* were highly increased in *P. infestans* infected leaves with late blight disease. Additionally, *StCNGC2*, *StCNGC8, StCNGC14*, *StCNGC15-2*, *StCNGC16*, and *StCNGC18* showed increased expression in leaves with *B. cinerea*-induced grey mold or *P. carotovorum*-induced blackleg infection compared to mock treatment. Given previous findings that silencing *StCNGC2/StDND1* confers broad-spectrum resistance to pathogens such as powdery mildew, late blight, and gray mold (Sun et al., 2016a, 2017), we hypothesize that StCNGC2 also plays a role in blackleg resistance.

### *StCNGC2*-silenced potato plants showed enhanced resistance to blackleg disease

**Figure 6:**
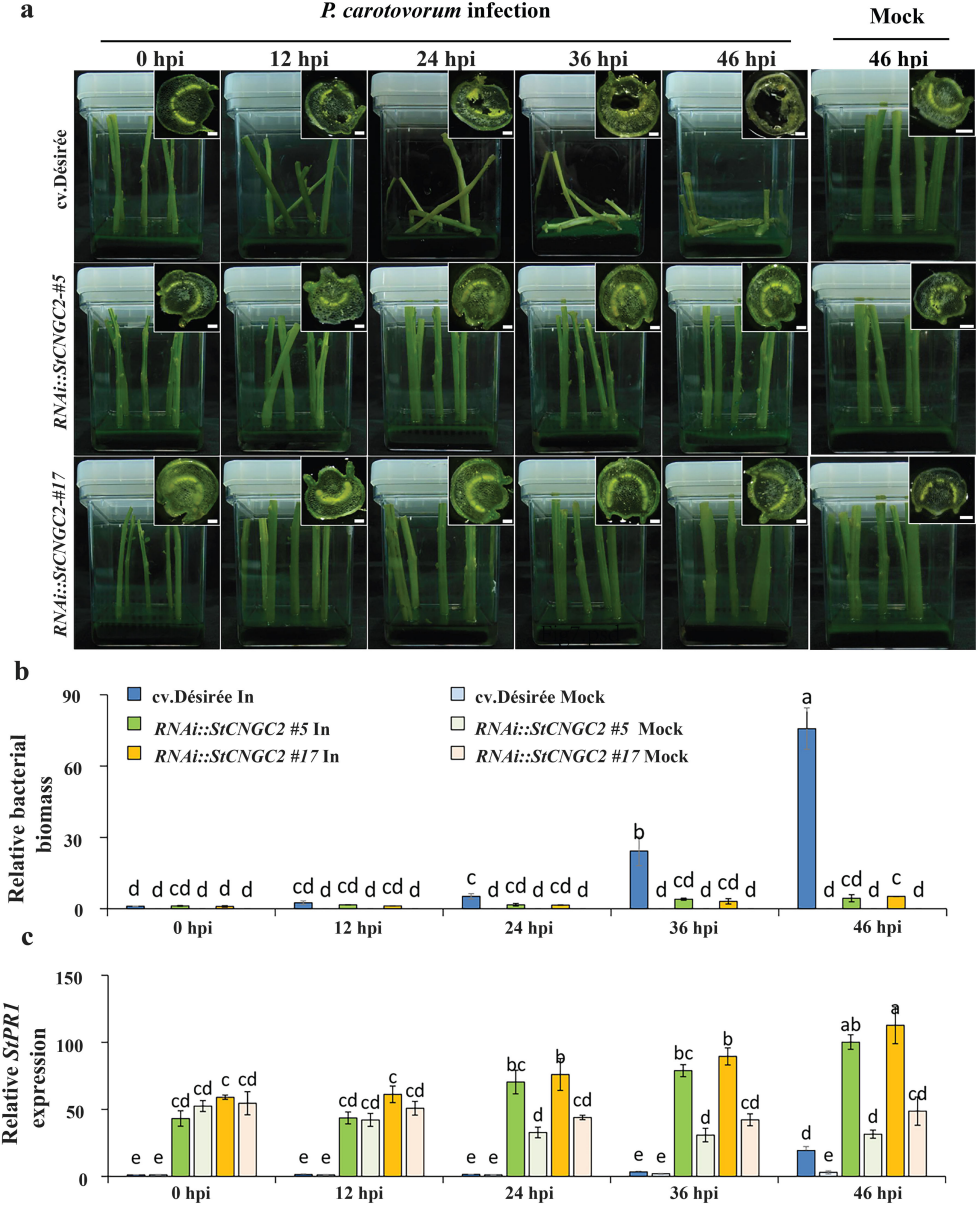
Analysis of potato blackleg inoculation in cv. Désirée- and *StCNGC2*-silenced potato plants. **(a)** Disease symptoms in potato petioles mock-treated or infected with *Pectobacterium carotovorum*. Pathogen-infected samples were imaged at 0, 12, 24, and 48 hours post-inoculation (hpi). The mock-treated sample was imaged at 48 hours post-inoculation (hpi) as a control. Preliminary observations indicated no visible changes in mock-treated samples throughout the time course. The insets show cross-section images of representative petioles at respective time points (Scale bar = 1 mm). **(b)** The relative bacterial content in mock treated and infected cv. Désirée- and *StCNGC2*-silenced potato plants. **(c)** The expression levels of *StPR1* genes analyzed by RT-qPCR. The different letters indicate significant differences between time points according to Duncan’s multiple range test (*P* < 0.05; n = 3) performed in SPSS. In: petioles inoculated by *P. carotovorum*; Mock: petioles immersed in MES buffer as a control.

Next, wild-type cv. Désirée and *RNAi::StCNGC2* potato plants were inoculated with *P. carotovorum*. Disease symptoms were observed at five time points (0, 12, 24, 36, and 46 hours post-inoculation [hpi]). In cv. Désirée plants, blackening of vascular tissue, and pulp degradation were evident by 12 hpi, accompanied by softening of petioles near the inoculation sites (**Figure 6a**). Later on, necrosis in the petiole pith increased progressively, leading to visible tissue degeneration and vascular blackening. In contrast, *RNAi::StCNGC2* plants did not show typical blackleg disease symptoms, such as wilting, soft rot, or vascular blackening, even after 46 dpi (**Figure 6a**).

To confirm the abovementioned observations, we quantified bacterial biomass by RT-qPCR at various times after inoculation with *P. carotovorum* (**Figure 6b**). In cv. Désirée plants, there was a steady increase in bacterial biomass from 12 hpi. In contrast, in *RNAi::StCNGC2* plants, the bacterial biomass remained restricted to a low level, confirming an increased resistance to *P. carotovorum* (**Figure 6b**).

Previously, elevated expression levels of *PR1*, a well-established marker of systemic acquired resistance (SAR), were reported in the respective mutants with reduced expression of *CNGC2* in *Arabidopsis*, potato and tomato (Clough et al., 2000; Sun et al., 2016b, 2017). To investigate the mechanism underlying the reduced *P. carotovorum* susceptibility in *StCNGC2*-silenced potato plants, we measured the expression of *StPR1* in a time-course infection (0, 12, 24, 36, and 46 hpi) by *P. carotovorum*. Noteworthy, a significantly higher *StPR1* expression was observed in *RNAi::StCNGC2 #5* and *#17* than in cv. Désirée even under mock treatment. When challenged with *P. carotovorum, StPR1* was significantly induced in cv. Désirée at 46 hpi, *StCNGC2-*silenced plants showed an earlier induction (at 24 hpi) (**Figure 6c**).

## Discussion

**Figure 7:**
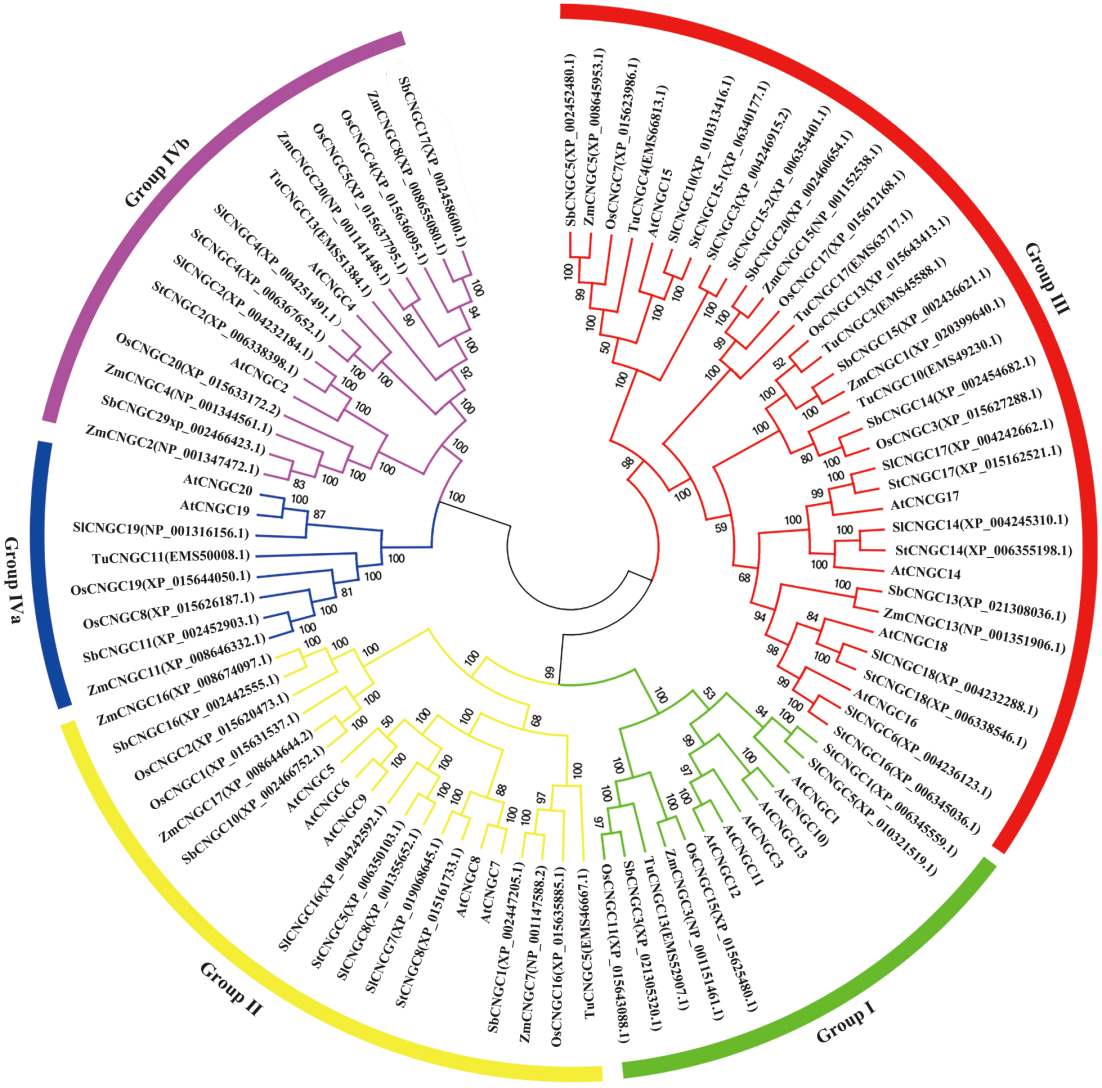
Phylogenetic analysis of the CNGC proteins in corn (ZmCMGC), rice (OsCNGC), wheat (TuCNGC), Sorghum (SbCNGC), *Arabidopsis* (AtCNGC), potato (StCNGC), and tomato (SlCNGC). The subgroups are marked by color bars. The phylogenetic tree was constructed using the neighbor-joining method with 1000 bootstrap replication.

Genome-wide studies of the *CNGC* family have been carried out in many plant species. In this study, we identified 11 *CNGC* family genes in potato. Additionally, 14, 8, 13, and 12 *CNGCs* were identified in rice, wheat, corn, and sorghum, respectively (**Figure 7**). The pI and charge of a protein are important for its solubility, subcellular localization, and interaction (Khaldi and Shields, 2011).

Studies indicate that cytoplasmic proteins exhibit an acidic isoelectric point (pI < 7.4), nuclear proteins display a more neutral pI (7.4 < pI < 8.1), and membrane proteins frequently possess basic pI values (pI > 8.1), with basic residues adjacent to the membrane-spanning region enhancing protein stabilization within the membrane (Schwartz et al., 2001; Khaldi and Shields, 2011). According to our results, the 11 StCNGCs contain varying numbers of transmembrane domains (**Supplementary Table S1**), and most of them (except for StCNGC8 and StCNGC18) have high pI values (> 8.1) (**Table 2**), indicating that they could be membrane proteins. The *StCNGC* genes showed high similarity to their corresponding *AtCNGC* genes in terms of plant CNGC-specific domains, amino acid composition, gene structure, and phylogenetic classification. They were categorized into five distinct groups with high bootstrap support. Homologous genes within the same group are expected to share comparable structural, functional, and evolutionary characteristics (Huang et al., 2018; Cheng et al., 2021; Wang et al., 2021b). Our finding offers insights into the roles of *CNGC* genes in potato.

**Table 2:** Description of potato *CNGC* family genes.

Gene name	Gene ID	RefSeq ID	Location	Strand	ORF(aa)	CDS(bp)	MW(da)	pI
*StCNGC1*	LOC102603937	XM_006345497.2	5:44712282-44718644	+	723	2172	83793	9.25
*StCNGC2*	LOC102595037	XM_006338336.2	2:45786407-45790505	+	708	2127	81471.9	9.54
*StCNGC4*	LOC102593350	XM_006367590.2	10:1723497-1730523	–	688	2067	80267.73	8.31
*StCNGC5*	LOC102596228	XM_006350041.2	12:2345839-2353176	–	692	2079	78709.82	9.05
*StCNGC8*	LOC102590601	XM_015306247.1	3:56646680-56652798	–	689	2070	79862.67	7.19
*StCNGC14*	LOC102579778	XM_006355136.2	8:44907823-44912083	+	727	2184	83853.48	8.95
*StCNGC15-1*	LOC102583901	XM_006340115.2	11:41469896-41474338	+	710	2133	81181.51	9.18
*StCNGC15-2*	LOC102587625	XM_006354339.1	9:5628981-5631704	+	659	1980	76036.04	8.98
*StCNGC16*	LOC102589750	XM_006344974.2	3:53961000-53966772	–	679	2040	78157.05	8.64
*StCNGC17*	LOC102606277	XM_015307035.1	7:1954668-1964196	+	720	2163	82732.16	8.94
*StCNG18*	LOC102586859	XM_006338484.2	2:46911057-46916119	+	690	2073	79694.35	6.42

CNGCs play a crucial role in regulating various biological processes, including growth and development, responses to environmental stresses, and plant defense mechanisms (Qi et al., 2010). This study showed that the *cis*-elements in the promoter region of plant *StCNGCs* were highly diverse, including various types of light response elements, hormone-related elements and environmental stress elements. This diversity aligns with the expression profiles observed in different conditions (**Supplementary Table S4**). In-depth gene expression analyses in this study highlighted the critical roles of *StCNGC* genes in potato growth, development, and stress responses (**Figure 5**, **Supplementary Table S5**). We analyzed *StCNGCs* expression in different tissues and found that most *StCNGCs* were expressed at high levels in flower tissues, which is consistent with the findings of previous research on these genes in *Arabidopsis* (Gao et al., 2016; Pan et al., 2019). For instance, a mutation in *AtCNGC18* causes a defect in pollen tube growth, resulting in curved, short, and often thin pollen tubes that are unable to enter the transport tube (Frietsch et al., 2007). In this study, the expression of *StCNGCs* in response to abiotic stresses (drought, heat, and cold) and biotic stresses (late blight, grey mold, and blackleg) was also explored (**Figure 5**). Notably, *StCNGC18* showed a higher expression than other genes under heat and cold stress, indicating its putative role in extreme temperature stress response. *StCNGC2* (*StDND1*), showed increased expression under various biotic stimuli, consistent with its proposed function in mediating Ca^2+^ influx during PTI activation. Noteworthy, *StCNGC15-2, StCNGC16*, and *StCNGC18* were also highly expressed in leaf tissue undergoing late blight, grey mold and blackleg infections, suggesting their potential implications in broad-range disease resistance. While these findings provide new insights into the role of *CNGC* genes in blackleg disease, further studies utilizing gene editing approaches (Zhang et al., 2021) are required to validate the specific functions of *StCNGC15-2*, *StCNGC16* and *StCNGC18* in disease resistance.

The *CNGC2* (*DND1*) mutation in *Arabidopsis*, tomato, potato, and apple improved plant resistance to resistant to a broad-spectrum of pathogens, including bacteria, fungi, and oomycetes (Govrin and Levine, 2000; Jurkowski et al., 2004; Young and Krougliak, 2004; Genger et al., 2008; Su’udi et al., 2011; Zhang et al., 2024b). In this study, we demonstrated that silencing *StCNGC2* improves blackleg disease resistance (**Figure 6**). Interestingly, overexpressing onion *CNGC2* homolog gene in *Nicotiana benthamiana* improves plant resistance to *Phytophthora nicotianae* by promoting ROS accumulation, suggesting a positive role against pathogens (Qin et al., 2024). This contradiction suggests that CNGC2 homologs may have different regulatory roles in plant immunity, depending on the plant species, developmental stage, and type of pathogen encountered. These differences highlight the complexity of calcium signaling in plant immunity and underscore the importance of species-specific functional validation of each gene family member.

*PR1* is reported to be a gene marker for systemic acquired resistance and exogenous SA treatment induces *PR* gene expression in many dicotyledonous plant species (Gal-On, 2007; Qu et al., 2024; Zhang et al., 2024a). Many studies have shown that the accumulation of SA in plants increases resistance to pathogens. For example, blackleg-resistant *Atcngc2* mutant accumulates higher levels of SA than wild-type *Arabidopsis* plants; overexpression of *OsWRKY13* in rice increased SA accumulation and enhanced resistance to bacterial blight (Ahn, 2007; Qiu et al., 2007). In this study, we found the level of *StPR1* gene expression in *StCNGC2* silenced lines was significantly higher than that in cv. Désirée (**Figure 6**), indicating the elevated *StPR1* levels are associated with improved plant resistance.

Disrupting plant S genes represents an alternative approach to achieve recessive, and potentially more durable, disease resistance (Garcia-Ruiz et al., 2021; Koseoglou et al., 2022). Our results confirmed *StCNGC2* as an S gene contributing to susceptibility to *P. carotovorum*. Given that mutation of S genes has been shown to enhance disease resistance across multiple plant species (Zaidi et al., 2018), further genome editing of *StCNGC2* to investigate its role in broader biotic stress responses and the potential crosstalk among these pathways represents a promising direction for future research.

## Conclusion

In summary, 11 *StCNGC* genes were comprehensively identified in the potato genome and classified into four clades based on phylogenetic analysis with *Arabidopsis CNGC* groupings. The motif composition of StCNGC proteins and the *cis*-acting elements in their promoter regions were analyzed in detail. The expression profiles of *StCNGC* genes revealed their differential expression across various tissues, and under multiple stress conditions, indicating their involvement in plant growth and stress responses. Notably, silencing *StCNGC2* enhanced resistance to blackleg disease, highlighting its role as a susceptibility (*S*) gene. Furthermore, our findings suggest that *StCNGC* genes play roles in both biotic and abiotic stress responses, highlighting their potential to enhance potato resistance to pathogens and environmental challenges. Collectively, this study provides a comprehensive overview of the *CNGC* gene family in the potato genome and establishes a foundation for further investigation into the mechanisms underlying *StCNGC* function and their applications in crop improvement.

## Data Availability

All relevant data are contained within the article. The original contributions presented in the study are included in the article/**Supplementary Material**. Further inquiries can be directed to the corresponding author(s).
