# Supplementary material for: Genome-wide identification of cyclic nucleotide-gated channel gene family in Solanum tuberosum and silencing of StCNGC2 provides resistance to Pectobacterium carotovorum
Source: Front Plant Sci. 2025 Jul 15;16:1614191. doi: 10.3389/fpls.2025.1614191 (PMC12303897; doi:10.3389/fpls.2025.1614191)

**Fig. S1 Expression levels of *StCNGC* genes in different tissues.**

Transcriptome data obtained from the Spud DB database illustrate gene expression patterns in leaf, tuber, root, stem, and flower samples. Expression levels are presented as log-transformed FPKM values.


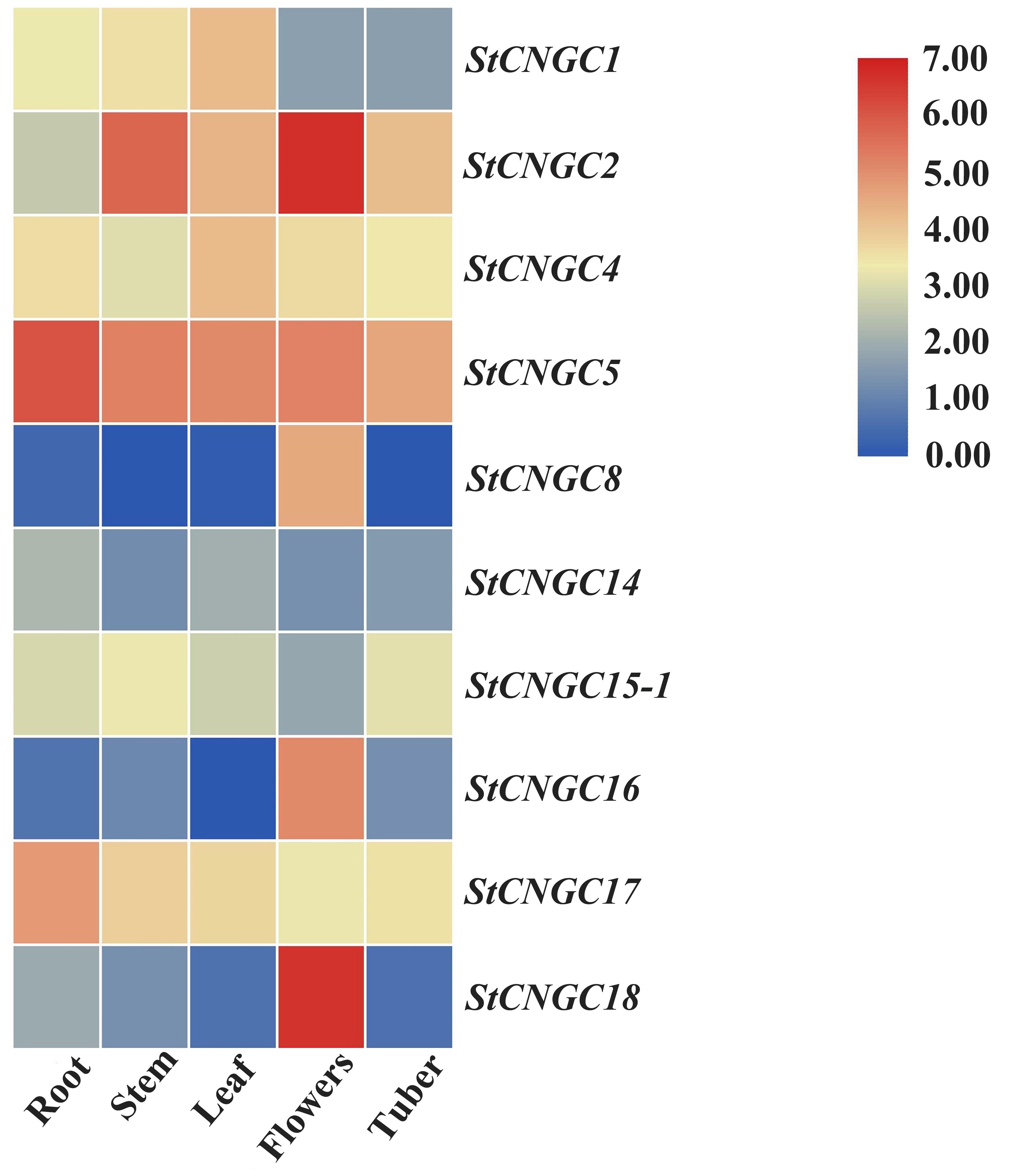


**Fig. S2 Whole protein sequence alignment of StCNGC family.**


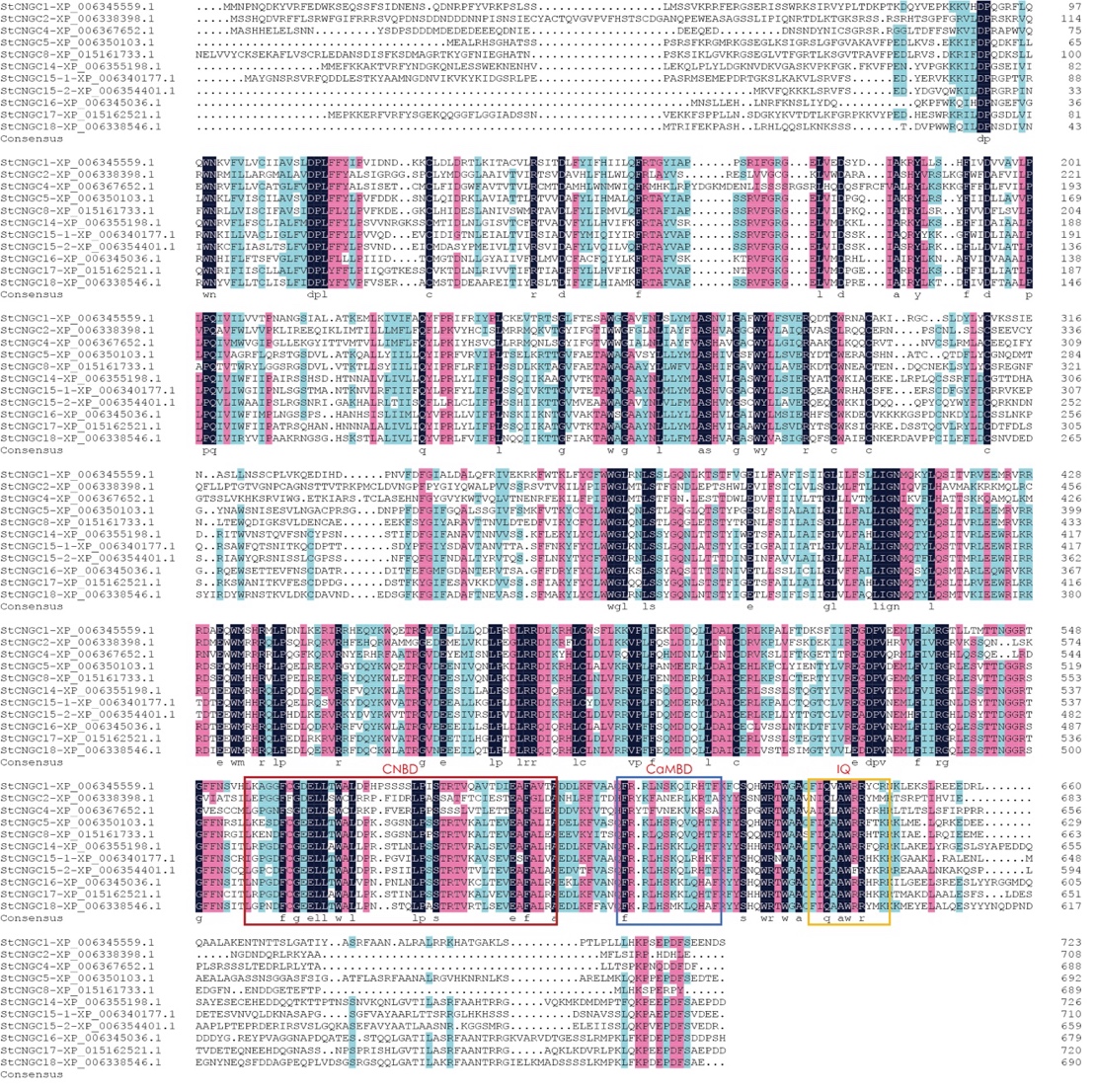

Supplement: Supplementary file 1 [file Supplementaryfile1.docx]
